# Randomized non-inferiority and safety trial of dihydroartemisin-piperaquine and artesunate-amodiaquine *versus* artemether-lumefantrine in the treatment of uncomplicated *Plasmodium falciparum* malaria in Cameroonian children

**DOI:** 10.1186/s12936-014-0521-2

**Published:** 2015-01-28

**Authors:** Akindeh M Nji, Innocent M Ali, Marcel N Moyeh, Eric-Oliver Ngongang, Aristide M Ekollo, Jean-Paul Chedjou, Valentine N Ndikum, Marie S Evehe, Guenter Froeschl, Christian Heumann, Ulrich Mansmann, Olumide Ogundahunsi, Wilfred F Mbacham

**Affiliations:** The Biotechnology Centre, University of Yaoundé I, Yaoundé, Cameroon; Department of Biochemistry, University of Yaoundé I, Yaoundé, Cameroon; Department of Biochemistry, University of Dschang, Dschang, Cameroon; Centre for International Health, Maximilians-University, Munich, Germany; Faculty of Medicine and Biomedical Sciences, University of Yaoundé 1, Yaoundé, Cameroon; Institute for Biometry and Epidemiology, Maximilians-University, Munich, Germany; Department of Statistics, Maximilians-University, Munich, Germany; Research Capacity Strengthening & Knowledge Management, WHO, Geneva, Switzerland

**Keywords:** Fever clearance, Parasite clearance, Adverse events, Non-inferiority, PCR-corrected

## Abstract

**Background:**

Artemether-lumefantrine and artesunate-amodiaquine are first-line treatment for uncomplicated malaria in Cameroon. No study has yet compared the efficacy of these drugs following the WHO recommended 42-day follow-up period. The goal of this study was to compare the clinical efficacy, tolerability and safety of artesunate-amodiaquine (ASAQ), artemether-lumefantrine (AL) and dihydroartemisinin piperaquine (DHAP) among children aged less than ten years in two malaria-endemic ecological regions of Cameroon.

**Methods:**

A three-arm, randomized, controlled, non-inferiority trial was conducted among children of either gender aged six months (>5 kg) to ten years (n = 720) with acute uncomplicated *Plasmodium falciparum* infection. Parents/guardians of children provided consent prior to randomization to receive ASAQ, DHAP or AL in the ratio of 2:2:1, respectively. Treatment outcome was assessed based on standard WHO 2003 classification after 42 days of follow-up. The primary outcome was PCR-corrected day-42 cure rates. The non-inferiority, one-sided, lower limit asymptotic 97.5% confidence interval (CI) on the difference in PCR-corrected cure rates of ASAQ and DHAP when compared to AL was accepted if the lower limit of the CI was greater than −10%. Secondary outcomes were parasite and fever clearances and day 7 haemoglobin changes.

**Results:**

PCR-corrected PP cure rates of 96.7, 98.1 and 96.3, respectively, for AL, ASAQ and DHAP was observed. The lower bound of the one-sided 97.5% CI calculated around the difference between day-42 cure rate point estimates in AL and ASAQ groups, AL and DHAP groups were, −6% and −4% respectively. There were no statistical significant differences in parasite or fever clearance times between treatments, although fever clearance pattern was different between ASAQ and DHAP. No statistical significant differences were observed in the occurrence of adverse events among treatment groups.

**Conclusion:**

ASAQ and DHAP are considered safe and tolerable and are not inferior to AL in the treatment of uncomplicated *P. falciparum* malaria in Cameroonian children.

**Trial registration:**

NCT01845701

## Background

Although malaria mortality rates have fallen globally in the Africa region [1], the disease continues to be a major threat to childhood health and development. The ministry of public health’s strategic plan for malaria control in Cameroon in 2012, reports that malaria is responsible for 31, 44 and 18 percent of all consultations, hospitalizations and deaths respectively, occurring in health facilities [2].

Efforts to curb malaria in the world have mainly been through vector control and chemotherapy. *Plasmodium falciparum* resistance to anti-malarials is a major drawback in the effective control of the disease. Anti-malarial drugs, such as chloroquine (CQ), sulphadoxine-pyrimethamine (SP) and amodiaquine (AQ), which were once very active against the malaria parasite, have been replaced by artemisinin combination therapy (ACT) in many malaria-endemic countries due to the emergence of resistance to these drugs [3]. A study in 12 sentinel sites in Cameroon (1999–2004) report CQ failure rates of 48.6% (irrespective of whether it was early or late failure) compared to low failure rates of 7.3 and 9% for AQ and SP, respectively. At the end of 2002 until 2004, AQ and SP were selected as first-line treatment and second-line treatment against falciparum malaria [4].

The association of a rapid acting and short elimination half-life anti-malarial drug such as artemisinin derivatives and a slower acting but longer half-life partner drug would provide the best combination to delay the onset of drug resistance [5]. The principal drugs recommended to slow the spread of resistance today are artemisinin-based combinations that include lumefantrine, piperaquine, AQ and SP as accompanying drugs. Some of these drugs, developed in Asia, were introduced into the African market without broad-based efficacy testing in different ecological sites. Cameroon adopted artesunate-amodiaquine (ASAQ) and artemether-lumefantrine (AL) as first-line treatments for falciparum malaria in 2006 [6]. No national evidence guided the choice of one artemisinin combination over the other.

A few studies have been conducted on comparative efficacy and safety of ACT in Cameroon. All evaluate efficacy within 28 days based on WHO 2003 guidelines for evaluating drug efficacy [7]. Whegang *et al*. [8] compared the efficacy of a comprehensive list of monotherapies and artemisinin combinations between 2003–2007 (including AL, ASAQ and dihydroartemisinin piperaquine (DHAP). Their results have shown generally high PCR-corrected cure rates with all forms of ACT (artemisinin combination therapy), with AL in the lead [8].

Despite the change in policy to ASAQ and AL as first-line drugs for uncomplicated *P. falciparum* malaria and the evidence of higher cure rates of AL over other forms of ACT reported in a 28-day follow-up, DHAP (Atekin®) is still sold in the drug stores in Cameroon [9]. The aim of this study is to investigate the efficacy of AL, ASAQ and DHAP during a 42 days follow-up period and to assess the non inferiority of ASAQ and DHAP when compared to AL in Cameroon children less than ten years of age.

## Methods

### Study sites

In order to minimize the evaluation bias due to differences in ecological regions, participants were recruited from Cameroon’s two distinct ecological regions (Mutengene in the Southwest region and Garoua in the North region). These are regions of low to moderate transmission.

Mutengene is situated at 04°01’N, 09°11’E with an Equatorial climate and 10,000 mm of rainfall per annum with an average temperature of 25°C. The vegetation is semi-mangrove and tropical wet forest. The study site is limited to the south and southeast by the sea and to the north and northeast by Mount Cameroon, an active volcano that is 4,100 m above sea level. The population works predominantly on palm and rubber estates owned by an agro-industrial complex known as the Cameroon Development Corporation (CDC). The women and children who do not work on these plantations engage in farm work and/or fishing.

Garoua lies at 06°24’N, 10°46’E, and serves as a river port in years when the rainfall is abundant. Situated in the River Benue basin, it receives an average annual rainfall of 380 mm. It has about four months of rainy season. Temperatures average 31°C for most of the year and the vegetation is guinea-savannah. The population is predominantly Muslim and comprised mainly of cattle raisers.

### Study design

This study was a three-arm, open-labelled, randomized, controlled, non-inferiority trial comparing the efficacy, safety and tolerability of ASAQ and DHAP to AL in children aged six to 120 months following a 42-day follow-up period.

### Sample size

The sample size for this study was calculated *a priori* the assumption that AL would have a cure rate above 94%. To demonstrate with 95% confidence (α = 0.05) that ASAQ or DHAP were acceptable if they are at worse 10% (d) inferior in the occurrence of failures, 10% risk (β) is accepted to rule out the null hypothesis of the lack of inferiority.

Using the formula with f (α, β) statistics: *N* (*sample size*) = [2p × (100 − p) × f(a, b)]/d^2^ [10]

$$ \begin{array}{c}\hfill =\left(2\times 94\times 6\times 10.5\right)/1{0}^2\hfill \\ {}\hfill =118\ \mathrm{in}\mathrm{dividuals}\ \mathrm{in}\ \mathrm{the}\ \mathrm{smallest}\ \mathrm{arm}\hfill \end{array} $$

Considering that other trials have reported loss to follow-up and withdrawal rates of 10% in 28 days’ follow-up, assuming a 20% loss to follow-up and withdrawal for 42 days’ follow-up was reasonable. This allowed that a minimum of 142 cases for the AL arm, and 284 cases for each of the two tested arms (Coarsucam® and Duo-cotecxin®) would be required, to make a total of 710 participants for all study sites. For the purposes of block randomization, a sample size of 720 was preferred.

### Study procedures

#### Participant enrolment

Children of either gender between the ages of six months and ten years with a minimum weight of 5 kg, meeting the inclusion criteria and who did not fulfil any of the exclusion criteria, were enrolled. Participants were enrolled if they were microscopically (using Giemsa-stained thick film) confirmed with acute uncomplicated *P. falciparum* malaria (1,000-100,000 parasites/μl), fever with an axillary temperature ≥37.5°C, ability to ingest tablets orally, and an assent from parents or guardians to attend the clinic on follow-up visits. Exclusion criteria included having any criteria for severe malaria, inability to drink or breastfeed, persistent vomiting, convulsion, unrousable coma, prostration, hypersensitivity to the study drugs, severe gastrointestinal disease or malnutrition (weight for height <70%), severe anaemia (haemoglobin <5 g/dl), and any other clinically severe condition as judged by the trial physician. The first patient was enrolled in 2009 and the final patient was enrolled in April 2013

#### Randomization

A randomization list was produced according to a randomization allocation schedule generated by a computer-based randomization program [11]. Allocation of participants was concealed in opaque envelopes that were opened sequentially by the study physician once consent was provided. The children were allocated DHAP, ASAQ or AL according to the ratio 2:2:1. The randomization number was recorded on the case report form as the study identification code and used in labelling all study-related laboratory samples.

#### Treatment and follow-up

AL (Coartem®: Novartis, Switzerland) tablets were provided in blister packs. Each tablet contains 20 mg artemether and 120 mg lumefantrine. Prescription was based on the weight of the child according to the recommended guidelines (5 to <15 kg (20/120 mg or one tablet), 15 to < 25 kg (40/240 mg or two tablets) and 25 to <36 kg (60/360 mg or three tablets) with second dose administered eight hours later and then twice daily for the subsequent two days. The AL was administered together with 200 ml of milk. ASAQ (Coarsucam®: Sanofi-Aventis, France) was available in three presentations and prescribed based on age groups and weights. Each participant received one dose in the arm of the co-formulated medication per day for three days (≥4.5 to < 9 kg (25 mg/67.5 mg) one tablet/dose; ≥9 to < 18 kg (50 mg/135 mg) one tablet/dose; ≥18 to < 36 kg (100 mg/270 mg) one tablet/dose. DHAP (Duo Cotecxin) was also a co-formulated medication presented as scored tablets of 40 mg dihydroartemisinin and 320 mg piperaquine phosphate each. Administration was based on recommended doses (2.25 mg/kg dihydroartemisinin and 18 mg/kg piperaquine per dose, rounded to the nearest half tablet) with each child receiving one dose per day for three consecutive days according to body weight. A child received a replacement dose if vomiting occurred less than 30 minutes after ingestion.

To ensure treatment compliance and to facilitate the supervised administration of the study drugs and full clinical and laboratory assessments and observation of early adverse effects, treatment administration was by direct observation on days 1, 2 and 3. All patients who completed their treatment were asked to report to the study clinic on days 7, 14, 21, 28, 35, and 42, or at any other time they felt unwell. Those who failed to adhere were visited by a community health worker and assisted to perform scheduled activities. Prior to drug administration, the study clinician made a clinical assessment of the patient and recorded all observations in the trials’ register and hospital patient file before transfer into the case report form. Abstracted data from hospital records were rechecked by a study site coordinator to ensure accuracy.

#### Laboratory analysis

Giemsa-stained thick blood films were prepared from capillary blood and examined under a microscope for determination of parasitaemia on all visit days and for all unscheduled visits. Parasitaemia was quantified by a standard approximation method (40 × the number of parasites per 200 leukocytes on thick film). Quality control of the microscopy readings was done by mask reading of 10% of the slides by a reference laboratory. Haemoglobin levels were measured on days 0, 7 and 42 and on any unscheduled visit using a HemoCue B-Hemoglobinometer (Hemocue®, Sweden). A full blood count, including differentials and biochemical parameters of liver and kidney function (alanine aminotransferase serum activity, bilirubin and creatinine serum concentrations), were investigated from in venous blood before treatment and on days 7 and 42 or on the day of re-appearance of parasitaemia. Any value that was found to be significantly greater than twice the upper normal limit was rechecked weekly until normal values were obtained. Blood samples were collected and spotted onto filter papers (3MM; Whatman®) for parasite genotyping on days 0, 14, 28, 35, 42 and during clinical or parasitological failure days. DNA (both for human genomic DNA and parasite) was extracted from blood spots on filter papers (Whatmann 3MM) by boiling in chelex-100 as described by Plowe *et al*. [12]. PCR analysis of polymorphic antigen markers including merozoite surface protein (MSP)-1, MSP-2 and glutamate-rich protein (GLURP) were used to distinguish parasites that were re-infecting from those that were recrudescent in all parasite recurrent infections during the period of follow-up. Recurrent parasite infections that contained both recrudescent and re-infecting parasites were considered recrudescent to simplify analysis.

#### Outcome assessment

Evaluation of therapeutic outcome was based on WHO guidelines on 42 days of follow-up [7]. The primary efficacy outcome was the proportion of patients with a PCR-corrected adequate clinical and parasitological response (ACPR) after a follow-up of 42 days. Based on these guidelines, ACPR was defined as the absence of parasitaemia on day 42 irrespective of axillary temperature, without previously meeting any of the criteria for early treatment failure (ETF), late treatment failure (LTF) or late parasitological failure (LPF). ETF was defined as development of danger signs of malaria on days 0, 1, 2 or 3 following ingestion of drug, with parasitaemia greater than day 0 value on day 2 or any parasitaemic on day 3 greater than 25% of the day 0 value irrespective of axillary temperature or any parasitaemia with axillary temperature ≥37.5°C on day 3. Late treatment failure is defined by the same guidelines as presence of parasitaemia with axillary temperature greater than 37.5°C between days 4 and 42 and without meeting any of the criteria for ETF. LPF was defined to be the presence of microscopic parasites on any day after day 7 with axillary temperature less than 37.5°C and without meeting any of the criteria for ETF and LTF. The cure rate was defined as the proportion of children with ACPR after due 42 days of follow-up.

The secondary efficacy end points of the study were to evaluate the efficacy of ASAQ, DHAP in comparison with AL on day 14 (D14) and day 28 (D28) and day 42 fever clearance and parasite times (FCT and PCT, respectively). FCT was defined as time (days) from the first dose until the first time (days) the body temperature decreases below and remained below 37.5°C for at least 48 hours, while PCT was defined as time (in days) from first dose until first total and continued disappearance of microscopic asexual parasitaemia for at least 48 hours. Therefore PCT and FCT were investigated on days 1, 2 and 3.

Safety was determined by comparing notified or reported adverse events (AEs) following treatment and during the follow-up period. An adverse event was defined according to the International Conference on Harmonization guidelines as any untoward medical occurrences in a patient administered a pharmaceutical product and which does not necessarily have a causal relationship to the treatment. Severe AEs are untoward medical occurrences that at any dose, result in death, are life threatening, require hospitalization, prolongation of existing hospitalization or result in persistent and significant disability, or is a congenital anomaly/birth defect.

#### Statistical analysis

Data were entered using Microsoft Office Access. Statistical software SPSS version 17 (Somers, NY, USA) was used for data management and processing. Data were analysed using the software ‘R’ version 2.11.1. The primary efficacy outcomes were analysed using both the intention-to-treat (ITT) and per-protocol (PP) data sets. The ITT population was made of all patients who received at least one dose of study drug. Participants who deviated from study protocol were included in the analyses. PP analyses included patients who adhered to the protocol until attainment of an end point or 42 days’ post-treatment. PCR-adjusted cure rates between treatments were compared using χ2 test as well as the odds ratios for likelihood of cure with 95% confidence intervals (CIs). Similarly, safety variables were analysed using ITT data. Comparison of categorical variables was analysed using χ2 test (or Fisher’s exact test as appropriate). Continuous data were tested for normality (Shapiro Wilk test). Normally distributed data were analysed using the t-test (or paired t-test where appropriate) or ANOVA and for non-normal data, Wilkoxon rank test and Kruskall Wallis test (for more than two groups) was used.

Non-inferiority of ASAQ and DHAP compared to AL was assessed by constructing a one sided, lower limit asymptotic 97.5% CI on the difference of PCR-corrected cure rates of ASAQ and DHAP when compared to AL. Non-inferiority was declared if the lower limit of the CI was greater than −10% (for ASAQ or DHAP minus AL). A P-value less than alpha = 0.05 was considered statistically significant in all the analyses.

#### Ethical considerations

Ethical clearance for the study was obtained from the Institutional Review Boards of the Cameroon Baptist Health Services, the Biotechnology Centre of the University of Yaoundé I, and the WHO Ethical Review Committee. A Data Safety and Monitoring Board appointed for the study met twice every year through the duration (2009-April 2013) of the study. The trial was monitored by a WHO/TDR consultant clinical trial monitor to ensure that all clinical trial activities were in compliance with Good Clinical and Laboratory Practices and approved study protocol. The trial was registered in the NIH clinical trial registry number NCT01845701.

## Results

### Patient flow and baseline characteristics

**Figure 1:**
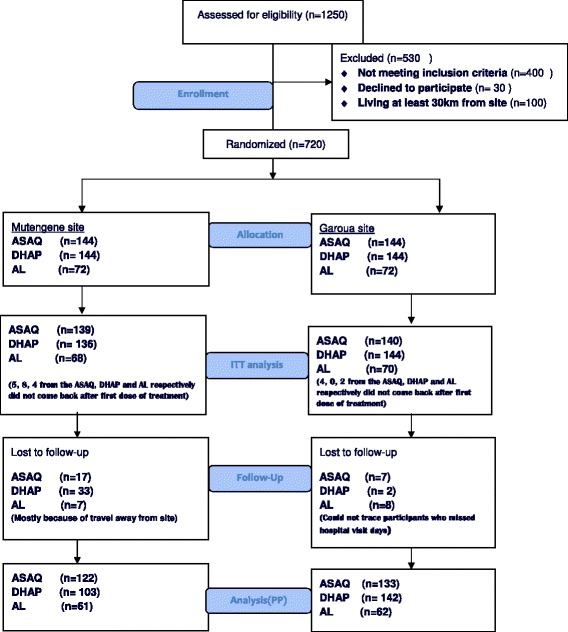
**The trial profile.**

In total, 1,250 children were screened from which 720 were randomized and enrolled into the three treatment arms (Figure 1). Of these 720, 23 declined immediately after taking their first dose of the trial drug and thus 697 treatment outcomes were available in the ITT. Another 74 patients were censored during the course of the 42 days, mainly due to loss to follow-up and protocol violation. Therefore, 623 outcomes were available in the PP analysis (86.5% protocol compliance). There was no statistical significant difference in baseline characteristics (Table 1) at enrolment between the three treatment arms.

**Table 1:** **Baseline characteristics of randomized study participants (ITT population)**

**Characteristics**	**ASAQ**	**AL**	**DHAP**	**P-value**
Age* (months) ± SD	55.35 ± 34.5	57.97 ± 33.8	54.88 ± 32.9	0.67
Weight* (kg) ± SD	16.9 ± 7.3	17.8 ± 7.7	16.9 ± 6.8	0.41
Axillary temperature* (°C) ± SD	38 ± 1.1	38 ± 1.1	37.8 ± 1.1	0.5
Haemoglobin* (g/dl) ± SD	10.4 ± 2.14	9.9 ± 2.1	9.9 ± 2.1	0.44
Parasite density** (/μl)	13,555 (1,040-100,000)	14,808 (1,060-100,000)	13,690 (1,040-100,000)	0.71
Creatinine (mg/l)	0.65 ± 0.46	0.73 ± 1	0.68 ± 0.72	0.48
ALAT (IU/l)	30 ± 52	25 ± 23	26 ± 28	0.81
Absolute neutrophil count /μl	47 ± 18	48 ± 19	47 ± 18	0.76
Sex (male: female)	(145:131)	(67:75)	(142:137)	0.5

* = mean ± SD (standard deviation); ** = median and range; ALAT = alanine aminotransferase.

### Primary outcome: efficacy of treatment and non-inferiority test

The AL, ASAQ and DHAP PCR adjusted cure rates in the ITT analysis population were 92, 91 and 89%, respectively (Table 2). The lower bound of the one-sided 97.5% CI calculated around the difference between day-42 cure rate point estimates in AL and ASAQ groups, AL and DHAP groups were, respectively (−0.05, 0.01) and (−0.04, 0.09). The lower bounds of these intervals are therefore greater than the pre-specified −10% non-inferiority margin. The drugs also had high cure rates in PP analysis populations. AL, ASAQ and DHAP had adjusted cure rates of 96.7, 98.1 and 96.3%, respectively (Table 2). No Statistical significant difference in PCR adjusted cure rates was observed between treatments. The lower bound of the one-sided 97.5% CI calculated around the difference between day-42 cure rate point estimates in AL and ASAQ groups, AL and DHAP groups were, respectively (−0.06, 0.03) and (−0.04, 0.05). Similarly, in the ITT analysis, the lower bounds of these intervals were as well greater than the pre-specified −10% non-inferiority margin.

**Table 2:** **Efficacy evaluation of AL(artemether lumefantrine), ASAQ (artesunate amodiaquine) and DHAP (dihydroartemisinin) in the treatment of** ***P. falciparum*** **malaria in Cameroon children**

	**ITT**			**PP**		
**Efficacy evaluation**						
**No PCR correction**	n	%	95% CI	N	%	95% CI
AL	138	80.4	72.6-86.4	123	91	84.2-95.2
ASAQ	279	81.4	76.1-85.7	255	89.4	84.8-92.7
DHAP	280	80.4	75.1-85.7	245	89.4	84.6-92.8
**PCR corrected**						
AL	138	92	85.9-95.7	123	96.7	91.3-98.9
ASAQ	279	91	89.3-95.7	255	98.1	95.2-99.2
DHAP	280	89	85.3-92.8	245	96.3	92.9-98.2

ITT = intention to treat population; PP = per protocol population.

Crude day-42 cure rates for AL, ASAQ and DHAP were similar (p = 0.9 and p = 0.75) for both ITT and PP analysis populations. The crude ITT percentage cure rates for AL, ASAQ and DHAP were 80.4% (95% CI, 72.6-86.4), 81.4% (95% CI, 76.1-85.7) and 80.4% (95% CI, 75.1-85.7), respectively. PP crude percentage cure rates were higher that the crude ITT cures with AL, ASAQ and DHAP having cure rates of 91% (95% CI, 84.2-95.2), 89.4% (95% CI, 84.8-92.7) and 89.4% (95% CI, 84.6-92.8), respectively. The PCR-corrected cure rates were generally higher than crude cure rates. AL, ASAQ and DHAP PCR-corrected percentage cure rates were 92% (95% CI, 85.9-95.7), 91% (95% CI, 89.3-95.7) and 89% (95% CI, 85.3-92.8), respectively for the ITT populations. PP PCR-corrected percentage cure rates were 96.7% (95% CI, 91.3-98.9), 98.1% (95% CI, 95.2-99.2) and 96.3% (95% CI, 92.9-98.2) for AL, ASAQ and DHAP, respectively.

### Secondary efficacy end points

#### Days 14 and 28 cure rates

Comparing therapeutic response outcomes for the different treatment arms did not show any significant difference for both day 14 (p = 0.51) and 28 (p = 0.78) end points, respectively (Table 3). No ETF was observed among patients in the AL and ASAQ arm. Patients on DHAP had a higher frequency of ETF and LCF.

**Table 3:** **PCR-adjusted therapeutic response (per protocol analysis) for day 14 and day 28**

	**Day 14**				**Day 28**			
**Outcome**	**AL**	**ASAQ**	**DHAP**	**P-value**	**AL**	**ASAQ**	**DHAP**	**p-value**
**ACPR**	119	250	236	0.51*	119	243	236	0.78*
**ETF**	0	0	2		0	0	2	
**LTF**	1	2	4		1	7	4	
**LPF**	3	2	3		3	4	3	

ACPR = adequate clinical and parasitological response; ETF = early treatment failure.

LCF = late clinical failure; LPF = late parasitological failure; AL = artemether-lumefantrine; DHAP = dihydroartemisinin-piperaquine; ASAQ = artesunate-amodiaquine; * = Fischer exact test (significant if there is at least one significant difference in therapy outcome level when comparing the three treatment regimen).

#### Parasite and fever clearance patterns

**Figure 2:**
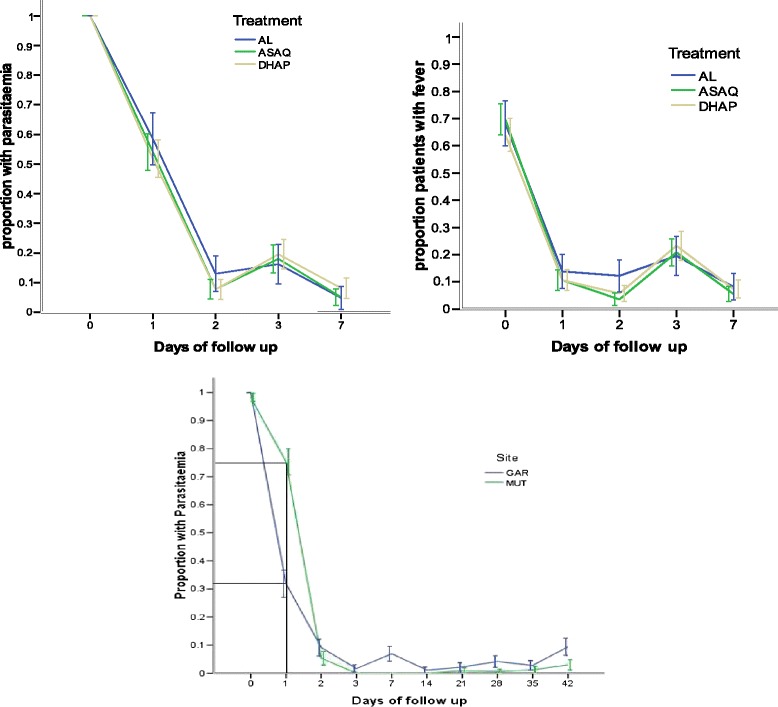
**Parasite and fever clearing patterns.**

Parasite clearance times were similar across the three study arms (Figure 2A). However there appeared to be a study site effect on parasite clearance, although not significant. Most participants in Mutengene site (75%) (Figure 2C), did not clear their parasites as measured by microscopy by the end of day 1 post-treatment compared to participants in Garoua (32%). Comparing the parasite clearance time across the different treatment arms and site did not show any significant difference by day 3 post-treatment. There was a difference in fever clearance patterns (Figure 2B) for the three treatment arms (Mantel cox pairwise comparison; p = 0.04) between ASAQ and DHAP. However, no significant difference (p = 0.21) was found between the treatment groups with respect to fever clearance time (FCT) at day 3.

#### Primary safety and tolerability outcomes

AEs frequency distribution among study participants (Table 4) show that vomiting, cough, rashes, and anorexia were slightly higher in the group of participants on the ASAQ and DHAP treatment arm. The drugs did not differ with respect to the type of AEs (all p values < 0.05). Although there was no significant statistical difference (P = 0.09) in the occurrence of all AEs when comparing the trial drugs, ASAQ (35.5%) and DHAP (37.9%) had higher number of AEs than AL (27.5%). One serious AE occurred involving a child who experienced severe fatigue after AL ingestion. The severe AE was resolved after a 3-day hospitalization and adequate clinical care.

**Table 4:** **Frequency of adverse events (mild to moderate-WHO grading) from days 1–7 after treatment initiation**

**Adverse event**	**AL**	**ASAQ**	**DHAP**	P value*
(Using ITT populations)	(N = 138)	(N = 279)	(N = 280)	
Abdominal pain--n(%)	5(3.6)	6(2.1)	13(4.60	0.26
Anorexia--n(%)	1(0.72)	8(2.9)	12(4.3)	0.13
Joint ache--n(%)	2(1.4)	2(0.71)	2(0.71)	0.71
Cough--n(%)	9(6.5)	13(4.7)	18(6.4)	0.61
Diarrhoea--n(%)	4(2.8)	8(2.8)	9(3.2)	0.96
Fatigue--n(%)	3(2.2)	7(2.5)	4(1.4)	0.65
Fever--n(%)	2(1.4)	6(2.2)	3(1.1)	0.58
Rash--n(%)	4(2.9)	18(6.4)	16(5.7)	0.31
Vomiting--n(%)	8(5.8)	31(11.1)	27(9.6)	0.21
**TOTAL--n(%)**	**38(27.5)**	**99(35.5)**	**106(37.9)**	**0.09**

*3 sample Chi-squared test for proportions with or without continuity correction as appropriate; ITT = intention to treat.

There was a slight decrease in mean haemoglobin levels in all treatment groups between days 0 and 7 (Table 5). However, the change was significant for DHAP (p = 0.0001) and AL (p = 0.003). Similarly there was a general slight increase in alanine transferase activity and creatinine levels in all treatment groups. Between treatments, no statistical significant difference was found comparing these parameters (Table 5).

**Table 5:** **Evolution of biological parameters with respect to AL, ASAQ and DHAP**

**Parameter**	**AL**				**ASAQ**				**DHAP**				
	**DO**	**D7**	**D0-D7**	P*	**DO**	**D7**	**D0-D7**	P*	**DO**	**D7**	**D0-D7**	P*	**P+**
**Hb (g/dl)**	10.2	9.56	0.46	**0.003**	10.4	9.92	0.48	**0.367**	9.92	9.37	0.55	**0.001**	**0.988**
**[SD]**	[2.2]	[1.93]	[1.67]		[6.90]	[5.63]	[8.55]		[2.15]	[1.82]	[1.79]		
**ALAT (IU/l)**	28.07	22.01	6.05	**0.114**	23.59	29.61	−0.61	**0.134**	25.75	35.33	−9.58	**0.288**	**0.355**
**[SD]**	[40.25]	[16.16]	[42.19]		[19.30]	[0.47]	[63.57]		[2.62]	[7.3]	[14.67]		
**CREA (mg/l)**	0.76	1.07	−0.31	**0.476**	0.65	0.68	−0.03	**0.565**	0.71	1.1	−0.39	**0.171**	**0.531**
**[SD]**	[1.04]	[4.74]	[4.84]		[0.03]	[0.06]	[0.88]		[0.72]	[4.46]	[4.52]		

P* = paired t test, P== F test from anova (comparing difference across three drugs); Hb = haemoglobin level.

[SD] = Standard deviation, ALAT = alanine aminotransferase, CREA = Creatinine level.

There was slight increase (but for patients in the AL treatment arm where alanine transferase activity dropped from 28.07 IU/L to 22.01 IU/L) in alanine transferase activity and creatinine levels by day 7 post-treatment irrespective of the treatment group. Abnormal biological values were not accompanied by persistent signs or symptoms suggestive of any serious AE.

## Discussion

Many studies are looking at the comparative efficacy of different forms of ACT in settings where the treatment is most likely to be used [13]. These studies seek to better inform malaria experts and health policy makers on the preferred ACT or alternatives [13] for different malaria-endemic countries [13-15]. In this study, the efficacy of ASAQ, DHAP and AL was compared in the same population during the same period 42 days after treatment administration. The drug had high cure rates for days 14, 28 and 42. These results are consistent with results from studies in other malaria-endemic countries in sub-Saharan Africa [14,16,17] indicating the choice to move to artemisinin-based combinations is appropriate in the face of emerging resistance to other common anti-malarials. The high cure rates are consistent in the two sites with different ecologies and climatic conditions. The high cure rates of these anti-malarial drugs and the effective use of insecticide-treated bed nets could significantly reduce morbidity and mortality in the Cameroonian population [18].

This study shows similar ACT cure rates in Mutengene and Garoua and supports the nationwide implementation of ACT irrespective of geographic location and ethnicity and brings an added advantage towards malaria elimination. The authorities in the Ministry of Health in Cameroon are fighting the illicit sale of medication by road vendors and unauthorized agents. There is still wide circulation of competing drugs to those enforced by the government for treating malaria [1]. The situation is worsened with stock-outs of anti-malarials at recognized distribution centres [19,20]. Patients are obliged to search for alternatives without proper information on the source of anti-malarials, their efficacy and tolerability. The non-inferiority of the study drugs compared to widely used AL will enable caregivers to make informed prescription decisions. The proper use of alternative, available, efficacious and safe drugs is helpful in delaying artemisinin resistance, which is beginning to emerge [9].

Along with reports of emergence of drug resistance to efficacious ACT, there is a need to closely monitor the efficacy and safety of these drugs. One way to monitor anti-malarial drug resistance in the absence of validated molecular markers and appropriate *in vitro* models is by analysing parasite clearance times [21]. Parasite clearance time curves represented by the proportion of patients that clear parasites with respect to the time from onset of treatment did not show any appreciable delay across the three study drugs. Although parasite clearance times were not measured by the standard definition, patients in Garoua had an advantage in their parasite clearance time when compared to Mutengene in all treatment arms in first two days during treatment. Mutengene, located in the Equatorial forest, has malaria transmission all year round whereas in Garoua transmission is seasonal and peaks within the rainy season. Differences in clearance rates by day 2 post-treatment therefore do not seem to be related to malaria exposure. The day 0 geometric mean parasite densities across treatment arms were comparable (Table 1). This may point to a well-known phenomenon, that the Fulani are more efficient at controlling parasites during early infection compared to non-Fulani [22,23]. The Fulani group predominates in the northern Sahelian regions of Cameroon, However, there is a clear need to further investigate parasite and fever clearance by the standard definition of clearance time to gain a proper picture of parasite dynamics following treatment in the two regions [22,24]. Difference in parasite susceptibility could be a plausible reason as well. The proportion of patients with a temperature below 37°C after the 3rd day of first treatment and who remain so for the next 48 hours are similar across the different study drugs. Patients in the ASAQ treatment group and DHAP treatment group cleared their fever quicker than those in the AL group three days after starting treatment (Figure 2B). This difference however only suggests that patients taking ASAQ and DHAP, compared to AL were relieved of the symptom much faster and this did not influence the outcome of treatment.

There were no significant changes in levels of kidney and liver function parameters measured (creatinine, alanine aminotransferase) between days 0 and 7 across the different treatment arms. Considering haemoglobin changes post-treatment, a significant difference was observed (Table 5) in the day 0 and day 7 haemoglobin levels of patients in the AL and DHAP treatment arms. However, as shown in Table 5, these changes were transient and all patients recovered by day 42 post-treatment without any effect on the cure rates achieved. These findings show that the drugs are well tolerated biologically as demonstrated in several other studies in malaria-endemic areas [25].

## Conclusion

ASAQ and DHAP were non inferior to AL in the treatment of uncomplicated P. falciparum malaria in Cameroonian children under ten years old with PCR-42 day corrected cure rates > 96%.
